# *In vitro* Probiotic Potential and Anti-cancer Activity of Newly Isolated Folate-Producing *Streptococcus thermophilus* Strains

**DOI:** 10.3389/fmicb.2018.02214

**Published:** 2018-09-19

**Authors:** Armin Tarrah, Juliana de Castilhos, Rochele Cassanta Rossi, Vinicius da Silva Duarte, Denize Righetto Ziegler, Viviana Corich, Alessio Giacomini

**Affiliations:** ^1^Department of Agronomy, Food, Natural Resources, Animals and Environment, University of Padova, Padua, Italy; ^2^Department of Nutrition, Universidade do Vale do Rio dos Sinos, São Leopoldo, Brazil; ^3^Department of Microbiology, Universidade Federal de Viçosa, Viçosa, Brazil

**Keywords:** hemolysis, folic acid production, cytotoxic activity, gastrointestinal resistance, probiotic, *Streptococcusthermophilus*

## Abstract

Most probiotic strains commercially available today are lactic acid bacteria. Within this functional group, *Streptococcus thermophilus* is a thermophilic species widely used as starter culture for a huge number of dairy products. Besides being rapid acidifiers, many *S. thermophilus* strains are able to produce and release folate during growth but, unfortunately, they are seriously impaired during passage through the human gastrointestinal tract. In this work, we studied eight *S. thermophilus* strains isolated from dairy environments in Italy, which already had shown good technological properties, to evaluate their possible probiotic potential and cytotoxicity against cancer cells *in vitro*. All strains were also evaluated for some health-related properties such as susceptibility to most common antibiotics, hemolytic activity, resistance to simulated gastrointestinal conditions, bile salts hydrolytic activity, production of folate, adhesion to HT-29 human colorectal adenocarcinoma cells and cytotoxic activity against cancer cells and production of biogenic amines. Results revealed that two fast acidifying *S. thermophilus* strains were found to possess *in vitro* probiotic properties along with anticancer activity and production of folate. These properties resulted similar and, in some cases, superior to those of *Lactobacillus rhamnosus* GG, a well-known commercial probiotic strain. These findings encourage further *in vivo* studies to evaluate the actual health benefits of these strains on the human host.

## Introduction

According to the WHO/FAO definition, probiotics are “live micro-organisms which, when ingested in adequate amounts, confer a health benefit on the host” (De Vrese and Schrezenmeir, 2008). During the last years, many lactic acid bacteria (LAB) have been studied for their probiotic potential, exploiting the Generally recognized as safe (GRAS) and Qualified presumption of safety (QPS) status possessed by some species. Many LAB are part of the normal microbiota of diverse districts of the human body and several others play fundamental roles in the production of fermented foods and for this reason are ingested in considerable amounts by consumers. Although *Bifidobacterium*, *Enterococcus*, *Lactococcus*, and *Pediococcus* genera include probiotic strains, most probiotic bacteria on the market today belong to the genus *Lactobacillus*. Unfortunately, most probiotics strains generally do not possess good technological characteristics and must therefore be added to fermented foods together with the technological strains. Although a number of probiotic strains are commercially available worldwide, the identification and characterization of new strains from different species is desirable as confirmed by many studies in this field published in recent years (Jeronymo-Ceneviva et al., 2014; Peres et al., 2014; De Paula et al., 2015; Oh and Jung, 2015).

As a member of LAB, *Streptococcus thermophilus* is identified as a thermophilic group of bacteria and it is used as starter in a great number of dairy products, thus being considered the second most important species of industrial LAB after *Lactococcus lactis*. It was estimated that over 10^21^ live bacteria are consumed annually by the human population as live cells, thus leading this species to achieve a market value of approximately 40 billion US$ (Iyer et al., 2010).

The technological use of *S. thermophilus* is mainly related to its capability to rapidly acidify the substrate, a particularly important feature, since it is known that a pH decrease induces modifications in bacterial (Maragkoudakis et al., 2013) and also yeast (Bovo et al., 2011, 2012) population composition. This is particularly relevant for aspects related to food safety, since most pathogenic bacteria grow very slowly or not at all at acidic pH. *S. thermophilus* is also well-known for production of folate, which is a necessary component of the human diet (Sybesma et al., 2003). Different LAB have been checked for their ability to produce folate and some fermented dairy products were reported to contain good amounts of this molecules, e.g., up to 110 μg of folate per liter in yogurt, due to the activity of LAB (Rao et al., 1984). Of the two species present in yogurt, *Lactobacillus delbrueckii* subsp. *bulgaricus* and *S. thermophilus*, only the latter is known to product folate. It has been reported that consume of food containing folate-producing bacteria is able to increase plasma folate concentration in humans (Rao et al., 1984). In addition, particular attention has been given to the role of probiotics in the reduction of invasion in cancer cells studies (Jiang et al., 2015). Beneficial effects of LAB in cancer therapy are not related only to their immunomodulatory effects. They have been indicated the expression of different genes involved in cell transformation, migration and invasion and it has to be noted that the anti-cancer properties of probiotics bacteria could be strains dependent (Motevaseli et al., 2017). Therefore, investigation of cytotoxic activity against different cancer cells could be very interesting feature of newly isolated bacteria.

The aim of this study was to select new potential probiotic *S. thermophilus* strains among a group of eight strains isolated in Italy from different dairy environments, which already had shown good technological properties. In particular, the genome of these eight strains had been sequenced and analyzed for the presence of genes related to bacteriocins and production of exopolysaccharides (Vendramin et al., 2017). Moreover, acidification kinetics (Vendramin et al., 2017), growth at different temperatures (Tarrah et al., 2018b) and at different pH values (Tarrah et al., 2018a), along with the growth dynamics using different energy sources (Tarrah et al., 2018c) were evaluated previously. Our work examined the existence of *S. thermophilus* strains with both good probiotic and technological properties. Since this species has enormous relevance as technological starter, the opportunity to take a good advantage from strains of this this multipurpose species for the food industry and human health contemporarily appears of great interest. Hence, we studied the capability to withstand the transit through the gastrointestinal tract and the ability to hydrolyze bile salts. The absence of hemolytic activity and of transmissible antibiotic resistance was also examined, along with the capability to produce biogenic amines such as histamine and tyramine. Finally, we looked for health-related traits, namely the production of extracellular folic acid (vitamin B9) and the ability to attach and inhibit the growth of human HT-29 colorectal adenocarcinoma cells.

## Materials and Methods

### Bacterial Strains and Standard Growth Conditions

The strains of *S*. *thermophilus* used in this work are listed in **Table 1**. *Lactobacillus rhamnosus* GG (ATCC 53103) was included in most tests as reference strain for probiotic properties. Streptococci were routinely grown at 37°C in M17 medium (Difco, United States) containing 0.5% lactose, unless otherwise stated. All bacteria were stored at -80°C in M17 containing 20% (v/v) glycerol. Each strain was sub-cultured three times in M17 broth prior to its use.

**Table 1:** Strains of *S. thermophilus* used in this study.

Strain	Geographical region	Isolation matrix	Animal	Reference
1F8CT	Veneto	Curd from raw milk	Cow	Treu et al., 2014b
MTH17CL396	Valle d’Aosta	Fontina cheese	Cow	Treu et al., 2014c
M17PTZA496	Valle d’Aosta	Fontina cheese	Cow	Treu et al., 2014c
TH982	Campania	Mozzarella curd	Buffalo	Treu et al., 2014b
TH985	Campania	Mozzarella whey	Buffalo	Treu et al., 2014b
TH1435	Friuli Venezia Giulia	Raw milk	Goat	Treu et al., 2014d
TH1436	Friuli Venezia Giulia	Raw milk	Goat	Treu et al., 2014d
TH1477	Veneto	Raw milk	Cow	Treu et al., 2014a


### Antibiotic Susceptibility Test

Antibiotic susceptibility tests were performed by using the agar overlay diffusion method, according to the National Committee for Clinical Laboratory Standards (Wayne, 2002). Fourteen antibiotics, commonly recommended by the European Food Safety authority (EFSA Panel on Additives and Products or Substances used in Animal Feed, 2008) were used, namely amoxicillin (10 μg), ampicillin (10 μg), cephalexin (30 μg), chloramphenicol (30 μg), ciprofloxacin (5 μg), cloxacillin (5 μg), erythromycin (15 μg), gentamicin (10 μg), kanamycin (30 μg), penicillin G (10 IU), streptomycin (10 μg), tetracycline (30 μg), trimethoprim (5 μg), and vancomycin (30 μg). All strains were cultured from the stock two times prior to assay in 10 ml of M17 broth, then they were incubated at 37°C for 24 h. Plates containing 16 ml of M17 medium were overlaid with 4 ml of M17 soft agar inoculated with 200 μl of overnight cultures to give a final concentration of about 10^7^ cells/ml in the overlay. After solidification, antibiotic disks (Liofilchem, Italy) were placed on the surface and plates were incubated at 37°C for 24 h. Finally, inhibition halo diameters were measured and compared to the values proposed by Charteris et al. (1998) to score strains as resistant, intermediate or susceptible. The test was performed in triplicate. *Escherichia coli* ATCC 25922 was used as quality control of the antibiotic disks.

### Hemolytic Activity Test

Fresh cultures of *S. thermophilus* strains were streaked on M17 plates containing 5% (w/v) of sheep blood (Thermo Fisher Scientific, United States), incubated at 37°C for 48 h and then checked for the presence of hemolytic haloes. *Staphylococcus aureus* ATCC 6538 and *Lb. rhamnosus* GG were included as positive and negative control, respectively (Pieniz et al., 2014). The experiment was repeated three times with three technical replicates each.

### Resistance to Simulated Gastrointestinal Conditions

The resistance of *S*. *thermophilus* strains to conditions simulating those of the gastro-intestinal tract was tested as previously described (Favarin et al., 2015) with the following modifications. The basic juice for the gastrointestinal assay contained (per liter) calcium chloride, 0.11 g; potassium chloride, 1.12 g; sodium chloride, 2.0 g; potassium dihydrogen phosphate, 0.4 g. It was sterilized by autoclaving at 121°C for 15 min. The artificial gastric juice, prepared 1 h prior to use, contained (per liter) 3.5 g swine mucin (Sigma-Aldrich, United States) and 0.26 g swine pepsin (Sigma-Aldrich, United States). The pH was adjusted to 2.5 with 1 N HCl, filter sterilized and then added to the gastrointestinal basic juice. Aliquots of 0.1 ml of bacterial cells suspensions obtained after three subcultures in M17 broth for 24 h were transferred to 0.9 ml of artificial gastric juice. After 1 h of incubation at 37°C with agitation at 200 rpm, microbial viability was evaluated by the micro drop technique. The medium for simulated intestinal conditions contained (per liter) 3 g Ox-bile extract (Sigma-Aldrich, St. Louis, MI, United States), 1.95 g pancreatin (Sigma-Aldrich, St. Louis, MI, United States) and 0.1 g lysozyme (Sigma-Aldrich, St. Louis, MI, United States). The pH was adjusted to 8.0 with 1 N sodium bicarbonate and the medium was filter sterilized. After gastric juice incubation, 1 ml of intestinal solution was added, and the incubation was continued at 37°C with agitation for further 3 and 5 h. Microbial viability was evaluated at each time point by the micro drop technique. The experiment was repeated three times with three technical replicates each.

### Production of Histamine and Tyramine

A defined decarboxylase medium was used (Mete et al., 2017) with some modifications. Five grams of tryptone, 8 g beef extract, 4 g yeast extract, 0.5 g tween-80, 0.2 g MgSO_4_, 0.05 g MnSO_4_, 0.04 g FeSO_4_, 0.1 g CaCO_3_, and 0.06 g bromocresol purple were dissolved in 1 l of deionized water and autoclaved at 121°C for 10 min. The pH of the medium was adjusted to 5.3 aseptically. Strains were grown in M17 broth overnight, washed three times with PBS and transferred to tubes containing the decarboxylase medium. After incubation at 30°C for 5 days, 200 μl of each culture were transferred to sterile tubes containing 2 ml of defined decarboxylase medium containing the specific amino acid L-histidine or L-tyrosine at 0.5% final concentration. All tubes were incubated for further 3 days at 30°C. The conversion of the color from yellow to purple was considered as positive response. The medium without amino acid addition was used as negative control. The experiment was performed with three technical replicates.

Furthermore, the eight *S. thermophilus* genomes (Vendramin et al., 2017) were inspected for the presence of genes involved in histamine and tyramine production by using BLASTn Megablast (Morgulis et al., 2008). For histamine, the histidine decarboxylation *hdc* cluster of *S. thermophilus* CHCC6483 (Accession number FN686790.1) was used, while for tyramine the *tdcA* gene of *S. thermophilus* 1TT45 (accession number FR682467) and *Lb. curvatus* HSCC1737 (Accession number AB086652) were chosen.

### Bile Salts Hydrolysis Activity

Fresh bacterial cultures were streaked onto M17 plates containing 0.5% taurodeoxycholic acid (Sigma-Aldrich, St. Louis, MI, United States). The hydrolytic activity was determined after 48 h of incubation at 37°C by inspecting the presence of a deoxycholic acid precipitation halo around positive colonies and into the surrounding medium. M17 plates without taurodeoxycholic acid were used as negative controls, whereas *Leuconostoc mesenteroides* SJRP 55 was used as positive control (Jeronymo-Ceneviva et al., 2014).

### Extracellular Folate Production

Folate production was quantified by using Folic Acid Casei medium (HIMEDIA laboratories, Mumbai, India) and *Lb.* *rhamnosus* ATCC 7469 as indicator strain (Horne and Patterson, 1988). Increasing amounts of folic acid determine a proportional increase in the growth of *Lb.* *rhamnosus* ATCC 7469. The indicator strain was prepared in advance by growing the strain in AOAC medium (Difco, United States) at 37°C for 24 h. After incubation, cultures were centrifuged, and the pellet washed twice with 10 ml of sterile 0.85% NaCl. Finally, cells were resuspended in 10 ml of 0.85% NaCl and diluted 1:100. Fifty-microliters aliquots were used to inoculate the assay tubes, prepared as follows.

The strains to be tested were grown in a chemically defined medium (Otto et al., 1983) without folic acid at 37°C for 6, 18, and 24 h. After centrifugation, 1 ml of the supernatant was added to a tube containing 5 ml of Folic Acid Casei medium and 4 ml of deionized water to give a final volume of 10 ml. Tubes were autoclaved at 121°C for 5 min, then cooled down at room temperature. Each tube was inoculated with 50 μl of *Lb.* *rhamnosus* ATCC 7469 suspension, prepared as described above. After incubation at 37°C for 24 h, the optical density was measured at 620 nm and the results interpreted according to the standard curve by considering the dilution factor of the supernatants. The standard curve was obtained according to the manufacturer’s instruction using 0.0, 0.1, 0.2, 0.4, 0.6, 0.8, and 1 ng of folic acid (Sigma-Aldrich, St. Louis, MI, United States) per assay tube (10 ml). The experiment was repeated twice with three technical replicates each.

### Adhesion to HT-29 Cells

Bacterial adhesion to HT-29 cancer cells was tested as previously described (Jacobsen et al., 1999), with the following modifications. HT-29 cells were grown in DMEM medium (Gibco BRL, United States) supplemented with 10% of heat-inactivated fetal bovine serum (Gibco BRL, United States) and 1% penicillin/streptomycin mixture (Gibco BRL, United States). Aliquots of 3 ml containing 1.5 × 10^5^ cells/ml were seeded on six-well Corning tissue culture plates and incubated at 37°C in 5% CO_2_ humid atmosphere until a complete monolayer was produced. The medium was changed every 48 h until a complete monolayer was formed. Then the medium was removed from the wells, plates were washed twice with sterile phosphate-buffered saline and filled with fresh antibiotic-free DMEM medium. Then plates were incubated at 37°C in 5% CO_2_ atmosphere for 30 min before adding the bacterial cells. The adherence test was performed by inoculating 120 μl of bacterial culture, suspended in antibiotic-free DMEM medium, at a concentration of about 1 × 10^8^ cfu/ml and incubating at 37°C for 3 h in 5% (v/v) CO_2_ atmosphere. After incubation, plates were washed four times with phosphate-buffered saline to release unbound bacteria. Fixation was carried out by adding 3 ml of methanol and incubating at room temperature for 10 min. Methanol was then removed and 3 ml of Giemsa stain solution (1:20) (Merck, Darmstadt, Germany) were added to the wells and again incubated at room temperature for 30 min to stain the cells. After staining, the wells were washed until no color was visible in the washing solution. Then plates were dried at 37°C and examined under an optical microscope at 1000× magnification. The adherent bacteria were counted in 20 random microscopic fields for each test. Bacterial strains were scored as non-adhesive when less than 40 bacteria were present in 20 fields, adhesive when containing 41–100 bacteria in 20 fields, and strongly adhesive when more than 100 bacteria were counted in 20 fields. The experiment was repeated three times with three technical replicates each.

### Cytotoxic Activity Against HT-29 Cells

The cytotoxic activity of *S. thermophilus* stains against HT-29 colorectal cancer cells was evaluated through the MTT [3-(4,5-dimethylthiazol-2-yl)-2,5-diphenyltetrazolium bromide] tetrazolium reduction assay (Mosmann, 1983) with minor modifications. Aliquots of 100 μl of HT-29 cell in DMEM medium containing 1.2 × 10^5^ cells/ml were introduced in the wells of 96-wells microplates. After 24 h of incubation, the supernatants were collected, adjusted to pH 7.0 with 1 N NaOH, lyophilized, and serially diluted in DMEM at the following concentrations: 125, 250, 500, 750, 1000, 2000, 4000, and 8000 μg/ml. When 50% confluence was reached, the medium was replaced with 100 μl of filtered supernatant from *S.* *thermophilus* cultures at different concentrations and cells were incubated at 37°C for 48 h under 5% CO_2_ atmosphere. After incubation, 20 μl of PBS containing 5 mg/ml MTT were added to each well and further incubated for 4 h. Successively, 100 μl of DMSO (Sigma-Aldrich, United States) were added to each well to dissolve formazan crystals by 20 min stirring at 200 rpm. MTT reduction absorbance was measured at 570 nm using a microplate reader (SpectraMax M5, Molecular Devices, United States). In addition, cells were incubated with M17 alone and with 3% DMSO that were used as negative and positive controls, respectively. This test was performed only on the best three strains on the basis of previous tests. The experiment was repeated two times with three technical replicates each.

### Statistical Analysis

Data were analyzed by one-way analysis of variance (ANOVA) and Tukey’s test was used as *post hoc* analysis. The IC_50_ (half maximal inhibitory concentration), which represents the dose necessary to inhibit half of the cells, was calculated by non-linear regression using the GraphPad Prism software (version 7, GraphPad Software, Inc., San Diego, CA, United States).

## Results and Discussion

### Strains Characteristics

In this study, eight strains of *S*. *thermophilus* newly isolated from dairy environments in Italy (**Table 1**) were studied to evaluate their potential probiotic properties. The genomes of these stains were previously sequenced and some genetic and metabolic characteristics had already been studied (Vendramin et al., 2017). In particular, all strains but 1F8CT were able to reach pH 5.2 within 24 h (Vendramin et al., 2017), and all grew at their best at 37°C, while TH1477 was the fastest at 42°C, and TH1436 at 34 and 30°C (Tarrah et al., 2018b). Regarding the influence of the initial pH of the growth substrate, all strains grew very well at 7.0 while in a medium at pH 6.0 some strains (1F8CT and TH985) were significantly affected and at pH 5.5 all strains grew much slower, although with different kinetics (Tarrah et al., 2018a). Regarding the use of different energy sources (Tarrah et al., 2018c), all strains were obviously able to use lactose, but only M17PTZA496, MTH17CL396, and TH1436 could use galactose. It has to be considered that galactose accumulation in foods can lead to some unfavorable events, such as cheese fractures due to CO_2_ overproduction by heterofermentative bacteria, browning on heat-treated foods such as Mozzarella in pizza preparation, and toxic effects on persons affected by galactosemia, a genetic disease involving galactose metabolism (Wu et al., 2015). Strain TH1435 was the only one unable to use glucose, while only 1F8CT, MTH17CL396, and TH1435 were able to use fructose. None of the strains were able to use xylose, the constituent of xylooligosaccharides (XOS) and inulin, that can be considered a positive feature since these prebiotic molecules, that can be added to the fermented food, may arrive intact to the intestine where are used by the gut microbiota. Finally, strains M17PTZA496, MTH17CL396, and TH982 resulted good potential EPS produces, a positive technological property for fermented dairy products (Vendramin et al., 2017).

### Hemolytic Activity Test

The lack of hemolytic activity is clearly one of the most important safety aspects to be considered for a food grade strain. Indeed, *in vitro* assessment of hemolytic activity on blood agar medium even for bacterial species that are considered GRAS is strongly recommended (FAO, 2002). None of the *S. thermophilus* strains under study showed β-hemolytic activity. All the strains were γ-hemolytic (i.e., without hemolytic activity) whereas *S. aureus* ATCC 6538, used as positive control, clearly showed β-hemolytic activity.

### Antibiotic Susceptibility Test

Investigation of antibiotic susceptibility is another important safety aspect regarding bacteria intended to be used in foods. Although the species *S. thermophilus* possesses the GRAS status, the presence of antibiotic resistance must be checked at strain level, since genes could have been acquired by horizontal gene transfer. It is therefore specifically required that they do not carry any transferrable antibiotic resistance genes that can be passed to pathogenic bacteria (Curragh and Collins, 1992). Contrary, intrinsic antibiotic resistance could be considered beneficial for the human host, to keep his gut microbiota alive during an antibiotic treatment (Charteris et al., 1998). Antimicrobial susceptibility data are reported in **Table 2**. All strains were susceptible to amoxicillin, ampicillin, cephalexin, chloramphenicol, erythromycin, penicillin G, tetracycline, and vancomycin, while all of them were resistant to streptomycin, kanamycin, and trimethoprim. According to previous studies (Ammor et al., 2007) and the guidelines by EFSA Panel on Additives and Products or Substances used in Animal Feed (2008), *S. thermophilus* strains are normally resistant to aminoglycosides antibiotics such as kanamycin, streptomycin, gentamicin, and trimethoprim. Therefore, this resistance is generally referred to as intrinsic and not able to be transferred horizontally. Regarding ciprofloxacin and cloxacillin, the strains showed different behavior: all were susceptible to ciprofloxacin and cloxacillin except TH1435 for ciprofloxacin and TH1435 and TH985 for cloxacillin, respectively, which evidenced intermediate resistance. These results indicate that the resistances found can be considered natural (intrinsic) and therefore not dangerous for human use, as confirmed by the presence of resistance to 6 out of 14 drugs tested that we found in the commercial strain *Lb. rhamnosus* GG (**Table 2**), in accordance to what was also reported in a previous study (Coppola et al., 2005).

**Table 2:** Antibiotic susceptibility of S. *thermophilus* strains measured as diameters (mm) of inhibition haloes.

Antibiotic	Amount (μg)	Strain								
		1F8CT	MTH17CL396	M17PTZAMT496	TH982	TH985	TH1435	TH1436	TH1477	GG
Amoxicillin	10	**32**	**32**	**33**	**32**	**28**	**28**	**29**	**34**	**21**
Ampicillin	10	**35**	**30**	**35**	**34**	**27**	**29**	**28**	**34**	**19**
Cephalexin	30	**31**	**30**	**32**	**31**	**32**	**29**	**26**	**33**	*12*
Chloramphenicol	30	**33**	**27**	**33**	**32**	***28***	**25**	**26**	**31**	**26**
Ciprofloxacin	5	**32**	**26**	**33**	**27**	**21**	18	**20**	**25**	15
Cloxacillin	5	**31**	**24**	**30**	**33**	17	17	**21**	**25**	17
Erythromycin	15	**25**	**28**	**33**	**32**	**29**	**28**	**24**	**33**	**21**
Gentamicin	10	*12*	**16**	**18**	**17**	**17**	**15**	*11*	**27**	*0*
Kanamycin	30	*11*	*6*	*12*	*14*	*12*	*10*	*6*	*11*	*0*
Penicillin G	10 IU	**34**	**35**	**33**	**36**	**34**	**30**	**31**	**33**	**27**
Streptomycin	10	*6*	*6*	*11*	*10*	*10*	*10*	*6*	*10*	*0*
Tetracycline	30	**30**	**27**	**22**	**33**	**28**	**27**	**26**	**32**	**22**
Trimethoprim	5	*6*	*6*	*6*	*6*	*6*	*6*	*6*	*6*	*0*
Vancomycin	30	**27**	**23**	**24**	**23**	**20**	**20**	**20**	**25**	*0*


*Susceptibility is indicated in bold, moderate susceptibility is underlined, and resistance is italicized. Cutoff values are taken from Charteris et al. (1998).*

### Production of Biogenic Amines

Biogenic amines produced by bacterial decarboxylation of amino acids can be found in many foods, particularly in fermented ones such as cheeses, wines, and beer (Halász et al., 1994). Although low levels of biogenic amines can be tolerated by humans, the ingestion of high amounts of these molecules, particularly histamine and tyramine, can cause food intoxication (Santos, 1996). For this reason, their production by a food-grade or probiotic strains should be low or absent. Regarding the strains studied in this work, the qualitative analysis performed on histamine and tyramine showed that only MTH17CL396 was able to produce both amines, while TH1436 produced tyramine only. The remaining six strains did not produce either of these two substances (**Supplementary Figure S1**) or could produce very low amounts that, according to (Bover-Cid and Holzapfel, 1999) are not be able to induce the color change to purple. From the limited number of studies on the production of biogenic amines by *S. thermophilus* (Gezginc et al., 2013; Ladero et al., 2015) it can be deduced that this characteristic is strongly strain dependent but, at least among the strains studied in this work, it appears to be a rare event.

We also performed an *in silico* analysis to look for genes related to biogenic amines production in the genomes of our strains. Results revealed that seven out of eight strains possess the histidine decarboxylation cluster *hdc* (**Supplementary Figure S2**) however, histamine production was detected only in *S. thermophilus* MTH17CL396. This outcome is in accordance with Gezginc et al. (2013), which observed a weak correlation between histamine production and presence of the *hdc* gene. Anyway, it must be remarked that histamine production in *S. thermophilus* can be influenced by environmental conditions, as demonstrated by Rossi et al. (2011) that found *hdc*A expression upregulated under specific conditions, such as 2% NaCl. They also reported production of histamine in the presence of broken bacterial cells or cell extracts containing *hdcA*, since the enzyme, if produced, could be released in the medium. In order to adequately examine such occurrences, a specific study would be needed. Concerning tyrosine, *tdcA* was not detected in any of the *S. thermophilus* strains used in this work. Notwithstanding, *S. thermophilus* MTH17CL396 and TH1436 were able to decarboxylate tyrosine *in vitro* (**Supplementary Figure S1**), indicating that other gene(s) could be involved in this pathway, as hypothesized for *hdcA* (Gezginc et al., 2013).

### Resistance to Simulated Gastrointestinal Conditions

**FIGURE 1:**
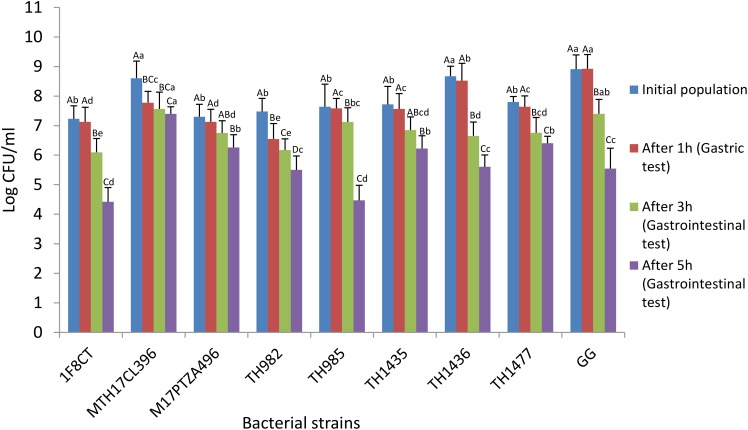
Survival of *S. thermophilus* strains and *Lb. rhamnosus* GG during exposure to *in vitro* simulated gastrointestinal conditions. Results are expressed as the mean ± SD (*n* = 3). Upper-case letters indicate significant differences among treatments and lower-case letters indicate significant differences among the strains (*P* < 0.05).

Survival during passage through the gastrointestinal tract is the key factor for probiotics to be able to benefit the host (Bezkorovainy, 2001). Recent studies reported conflicting data regarding the probiotic potential of *S. thermophilus*. Some authors are still discussing the viability of *S. thermophilus* after passage through the gastrointestinal tract. Indeed, it is well known that probiotic characteristics are strictly strain specific and this gives a strong motivation to keep seeking better strains (Senok et al., 2005). In our study, the resistance to simulated gastrointestinal conditions was investigated by incubating the cells for 1 h in gastric juice followed by 3 and 5 h in intestinal juice. The reference strain *Lb. rhamnosus* GG did not show any significant reduction in viability (**Figure 1**), but also *S. thermophilus* strains evidenced good resistance to gastric juice since reduction in viability was less than 1 log for most strains. Regarding the incubation in intestinal juice, following gastric passage, after 3 h strains MTH17CL396, M17PTZA496, TH985, and TH982 showed very good resistance while strain TH1436 had the lowest viability. Regarding the prolonged 5-h incubation, strains MTH17CL396, M17PTZA496, and TH982 confirmed very good viability levels, while TH985 had a dramatic decrease. In comparison with the commercial *Lb. rhamnosus* GG, most *S. thermophilus* strains showed just a slightly lower resistance to the gastric incubation test (**Figure 1**, brown bars), but *Lb. rhamnosus* appeared much less resistant to the intestinal conditions (1.5-log decrease), especially during prolonged incubation (3.4-log decrease), with respect to the best *S. thermophilus* strains MTH17CL396 (1.2-log decrease), M17PTZA496 (1.0-log decrease) and TH982 (2.0-log decrease). According to our results, some *S. thermophilus* strains showed better performances in comparison with the commercial *Lb. rhamnosus* GG strain regarding resistance to prolonged simulated intestinal conditions, particularly in the case of MTH17CL396, M17PTZA496, and TH982. Some authors reported that they were not able to recover *S. thermophilus* from human feces (Pedrosa et al., 1995) while, on the other hand, Brigidi et al. (2003) were able to recover *S. thermophilus,* during 6 days in a row, from fecal samples from 10 healthy subjects who had taken a pharmaceutical preparation orally for 3 days. Another study (Mater et al., 2005) strongly confirmed a significant recovery of viable *S. thermophilus* in human feces after yogurt consumption.

### Bile Salts Hydrolytic Activity

None of the tested *S. thermophilus* strains revealed bile salt hydrolytic (BSH) activity when grown on M17 agar containing 0.5% taurodeoxycholic acid. BSH activity of probiotic bacteria has been a controversial debate during the last decades. Although BSH is somehow related to intestinal survival of probiotics and cholesterol lowering in the human host, however, it cannot be considered as a desirable property for probiotics, since de-conjugated bile salts could have many undesirable effects for the human host (Berr et al., 1996; Mamianetti et al., 1999).

### Extracellular Folate Production

**FIGURE 2:**
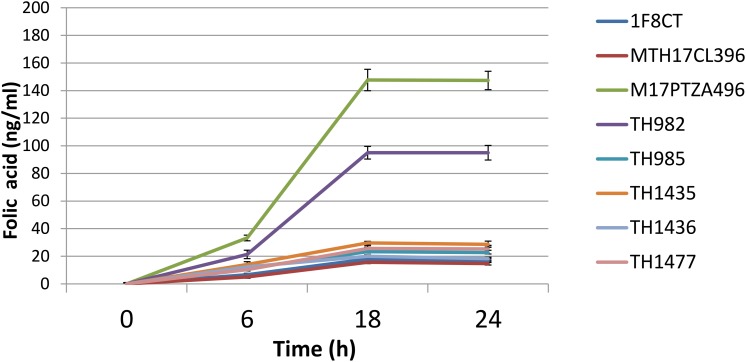
Kinetics of folate production detected in the medium during the growth of *S. thermophilus* strains. Error bars represent the standard deviation of each mean.

The capability to produce folate is of great interest for a potential probiotic strain, since it has been reported that consume of folate-producing bacteria can increase plasma folate concentration in humans (Valentini et al., 2015). Folate is an important factor in the human diet, being involved in essential functions of cell metabolism such as DNA replication, repair, and methylation and synthesis of nucleotides. Several studies reported that folate deficiency is quite widespread among people, particularly in women (Konings et al., 2001). The recommended daily intake in adult has been reported differently from place to place ranging from 200 μg in Europe to 400 μg in the United States (Sybesma et al., 2003). Recently, some authors claimed that high-folate diets protect against cardiovascular diseases (Boushey et al., 1995) and even against some forms of cancer (Ames, 1999). However, different LAB species and strains can have very different capabilities in folate production. While lactobacilli generally do not produce folate with the exception of *Lb. plantarum*, *L. lactis*, and *S. thermophilus* are considered good sources for production of folic acid (Crittenden et al., 2003; Sybesma et al., 2003). Extracellular folate concentration was monitored after 6, 18, and 24 h of bacterial growth to describe the trend of its production in the studied strains. Analysis on growth media revealed that all strains increased the amount of folate during growth (**Figure 2**). It should be noted that the highest amount of folate was measured after 18 h, which represents the late exponential phase. After that point, no significant differences were detected, and folate content remained constant. Extracellular folate production ranged from 5.06 to 147.67 ng/ml. Strains M17PTZA496 and TH982 showed the highest values, i.e., 147.67 and 95 ng/ml, respectively that was higher than that reported in literature (Sybesma et al., 2003).

### Adhesion to HT-29 Cancer Cells

**FIGURE 3:**
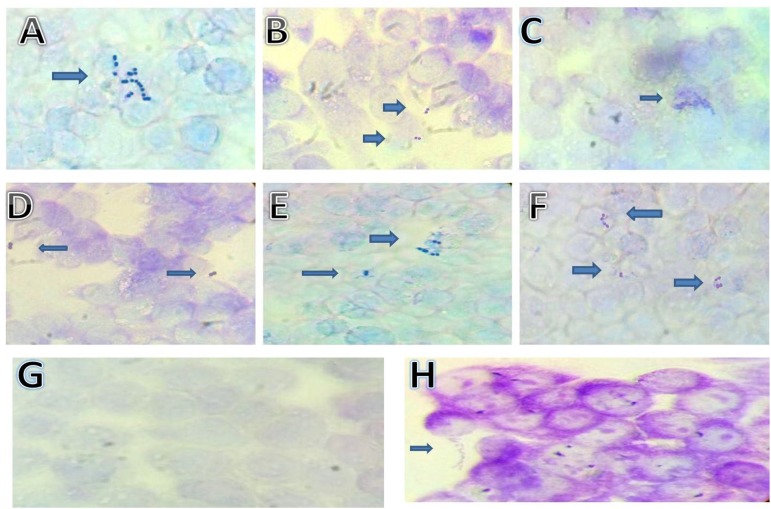
Adhesion of *S. thermophilus* cells on HT-29 cell cultures observed under light microscope (100×). **Strains: (A)** *S. thermophilus* MTH17CL396, **(B)** *S. thermophilus* M17PTZA496, **(C)** *S. thermophilus* TH982, **(D)** *S. thermophilus* TH985, **(E)** *S. thermophilus* TH1435, **(F)** *S. thermophilus* TH1436, **(G)** Blank HT-29 cell line, **(H)** *Lb. rhamnosus* GG.

The capability of bacteria to attach to the intestinal cells is another important key factor for probiotic microorganisms (Piatek et al., 2012). Images of *S. thermophilus* strains attachment to HT-29 colorectal cancer cells are shown in **Figure 3** and data on adherence are reported in **Table 3**. Strains MTH17CL396, M17PTZA496, TH982, TH985, TH1435, and TH1436 were strongly adhesive while the remaining showed a non-adhesive character. Moreover, strains MTH17CL396, M17PTZA496, TH982, and TH985 showed no significant difference (*P* < 0.05) in adhesion score with respect to *Lb. rhamnosus* GG while *S. thermophilus* TH1435 and TH1436 showed a significantly (*P* < 0.05) higher adhesive ability with respect to the reference strain. Adhesion ability of *S. thermophilus* strains was reported in several studies (Khali, 2009; Thomas et al., 2011). High cell surface hydrophobicity and production of extracellular polysaccharides were the main reasons for this characteristic in bacteria (Ruas-Madiedo et al., 2006; Tallon et al., 2007). In this respect, a previous study (Vendramin et al., 2017) reported that strains MTH17CL396, M17PTZA496, and TH982 are good producers of exopolysaccharides. On the other hand, many authors reported that survival in the feces following oral administration may be directly linked to colonization of the intestine by attaching to the epithelium (Lick et al., 2001; Brigidi et al., 2003; Salminen and Isolauri, 2006). Another study (Thomas et al., 2011) revealed that the presence of lactose enhanced the fermentative activity of *S. thermophilus* leading to higher level of luminal lactate which subsequently acts to modulate the host epithelium. Therefore, activation of enzymes involved in carbohydrate metabolism constitutes the metabolic signature of *S. thermophilus* in the GIT and favors the interaction with the colon epithelium.

**Table 3:** Adhesion potential of bacterial strains.

Strain	Adhesion score	Category
*S. thermophilus* 1F8CT	14.8 ± 2.3^c^	Non-adhesive
*S. thermophilus* MTH17CL396	383.9 ± 8.0^b^	Strongly adhesive
*S. thermophilus* M17PTZA496	363. 3 ± 8.5^b^	Strongly adhesive
*S. thermophilus* TH982	500.3 ± 6.0^b^	Strongly adhesive
*S. thermophilus* TH985	456.1 ± 7.6^b^	Strongly adhesive
*S. thermophilus* TH1435	506.1 ± 8.1^b^	Strongly adhesive
*S. thermophilus* TH1436	1062.3 ± 9.1^a^	Strongly adhesive
*S. thermophilus* TH1477	11.6 ± 1.9^c^	Non-adhesive
*Lb. rhamnosus* GG	420.8 ± 8.1^b^	Strongly adhesive


*Scores are the average number of adhering cells in 20 microscopic fields ± SD (n = 3). Numbers with same letters do not differ significantly (P < 0.05).*

### Cytotoxic Activity Against HT-29 Cell Line

The three strains that evidenced the most interesting characteristics during the tests described above and that have also technological potential according to previous studies (Vendramin et al., 2017), namely MTH17CL396, M17PTZA496, and TH982 were tested for cytotoxic activity against colorectal adenocarcinoma (HT-29) cells, using *Lb. rhamnosus* GG as reference strain. Results reveal that HT-29 cells were significantly inhibited by *S. thermophilus* MTH17CL396, M17PTZA496, and TH982 compared to the untreated cancer cell (**Table 4**). For all concentrations, no significant difference (*P* < 0.05) was found between *S. thermophilus* M17PTZ396 and *Lb. rhamnosus* GG when evaluated in a multiple comparison test (Tukey’s test). This was also confirmed by the determination of the IC_50_ values against HT-29 cells that resulted 1.42 ± 0.12 mg/ml for *Lb. rhamnosus* GG and 0.9 ± 0.13 mg/ml for *S. thermophilus* M17PTZ396, not significantly different, while values from *S. thermophilus* M17PTZA496 and TH982 were lower in comparison to *Lb. rhamnosus* GG. Overall, after 48 h of incubation, all three *S. thermophilus* strains indicated good antiproliferative effect on HT-29 cancer cells (**Table 4**). To exclude that such activity could be simply due to the lactic acid produced by all the bacteria tested, it is worth noticing that strain MTH17CL396, which displayed the best anticancer activity, has the worst acidification capability among the *S. thermophilus* strains tested, as previously reported (Vendramin et al., 2017). The inhibitory effect of *S. thermophilus* on HT-29 cells was previously demonstrated (Ewaschuk et al., 2006; del Carmen et al., 2015). Different mechanisms have been reported as to how LAB can inhibit colon cancer, which include enhancing the host’s immune response, binding and degrading carcinogens, producing antimutagenic compounds, and altering the physiochemical conditions in the colon (Hirayama and Rafter, 2000; Kumar et al., 2012). Moreover, probiotic bacteria are able to decrease the level of some dangerous enzymes in the human body such as glycosidase, β-glucuronidase, azoreductase, and nitroreductase which convert the precarcinogens into active carcinogens (Goldin, 1990; Pedrosa et al., 1995).

**Table 4:** Cytotoxic effect of different concentrations of lyophilized supernatant of *S. thermophilus* cultures, expressed as percentage of HT-29 cancer cells remained viable after 48 h of incubation.

Supernatant concentration (μg/ml)	M17PTZA396	M17PTZA496	TH982	GG	M17 growth medium
125	75.0 ± 0.02	93.7 ± 0.02	98.3 ± 0.01	65.8 ± 0.01	98.2 ± 0.05
250	65.2 ± 0.04	78.0 ± 0.07	71.1 ± 0.03	63.9 ± 0.09	86.9 ± 0.06
500	58.4 ± 0.12	65.3 ± 0.05	71.0 ± 0.05	58.9 ± 0.02	88.3 ± 0.11
1000	45.5 ± 0.07	62.5 ± 0.04	58.5 ± 0.01	54.1 ± 0.02	84.7 ± 0.06
2000	43.4 ± 0.05	61.8 ± 0.01	58.7 ± 0.05	48.2 ± 0.02	89.2 ± 0.05
4000	40.2 ± 0.03	58.8 ± 0.02	54.7 ± 0.04	41.1 ± 0.03	90.5 ± 0.05
8000	37.6 ± 0.03	57.6 ± 0.01	46.0 ± 0.04	29.1 ± 0.02	88.8 ± 0.24


*Lb. rhamnosus GG is the commercial reference probiotic strain. Growth medium M17 represents the negative control. A solution of 3% DMSO was used as positive control, which gave 26.2 ± 0.04 cell viability. Data are expressed as means ± SD.*

## Conclusion

The results of this study revealed that two strains of *S. thermophilus,* namely M17PTZA496 and TH982 possess very interesting *in vitro* probiotic properties along with anticancer activity and folate production. These two newly isolated strains showed potential characteristics similar and, in some cases, superior to the well-known commercial probiotic strain *Lb. rhamnosus* GG. We believe that these two strains of *S. thermophilus* could be contemporarily used as good probiotics and starter strains, considering their technological properties. Although strain MTH17CL396 did not show relevant production of folic acid, and produces tyramine and histamine, it has shown a very high cytotoxic effect against cancer cells. Therefore, further assessment by *in vivo* studies to evaluate potential health benefits in humans is recommended for these strains. The presence of probiotic properties in *S. thermophilus* strains are of particular interest, since, differently from most probiotics on the market, this species has enormous relevance as technological starter used in large amounts for the production of cheeses and fermented milks. Overall, we could take a good advantage from this multipurpose species for both the food industry and human health.

## Author Contributions

AT drafted the manuscript, performed the antibiotic susceptibility tests, hemolytic activity, bile salts hydrolysis activity, biogenic amines production and folate production by strains. AT and JdC performed the adhesion ability to HT-29 human epithelial cell and anticancer activity. RR and DZ participated in discussion and paper writing. AT and VD performed the resistance to simulated gastrointestinal juice. VD performed the genomic analyses related to tyramine and histamine production. VC and AG conceived and designed the experiments and revised the manuscript. AG supervised the project. All authors discussed the results and commented on the manuscript.

## Conflict of Interest Statement

The authors declare that the research was conducted in the absence of any commercial or financial relationships that could be construed as a potential conflict of interest.

## References

[B1] Ames B. N. (1999). Micronutrient deficiencies: a major cause of DNA damage. *Ann. N. Y. Acad. Sci.* 889 87–106. 10.1111/j.1749-6632.1999.tb08727.x 10668486

[B2] Ammor M. S. Belén Flórez A. Mayo B. (2007). Antibiotic resistance in non-enterococcal lactic acid bacteria and bifidobacteria. *Food Microbiol.* 24 559–570. 10.1016/j.fm.2006.11.001 17418306

[B3] Berr F. Kullak-Ublick G. Paumgartner G. Munzing W. Hylemon P. B. (1996). 7 alpha-dehydroxylating bacteria enhance deoxycholic acid input and cholesterol saturation of bile in patients with gallstones. *Gastroenterology* 111 1611–1620. 10.1016/S0016-5085(96)70024-0 8942741

[B4] Bezkorovainy A. (2001). Probiotics: determinants of survival and growth in the gut–. *Am. J. Clin. Nutr.* 73 399s–405s. 10.1093/ajcn/73.2.399s 11157348

[B5] Boushey C. J. Beresford S. A. A. Omenn G. S. Motulsky A. G. (1995). A quantitative assessment of plasma homocysteine as a risk factor for vascular disease: probable benefits of increasing folic acid intakes. *JAMA* 274 1049–1057. 10.1001/jama.1995.03530130055028 7563456

[B6] Bover-Cid S. Holzapfel W. H. (1999). Improved screening procedure for biogenic amine production by lactic acid bacteria. *Int. J. Food Microbiol.* 53 33–41. 10.1016/S0168-1605(99)00152-X 10598112

[B7] Bovo B. Giacomini A. Corich V. (2011). Effects of grape marcs acidification treatment on the evolution of indigenous yeast populations during the production of grappa. *J. Appl. Microbiol.* 111 382–388. 10.1111/j.1365-2672.2011.05060.x 21615635

[B8] Bovo B. Nardi T. Fontana F. Carlot M. Giacomini A. Corich V. (2012). Acidification of grape marc for alcoholic beverage production: effects on indigenous microflora and aroma profile after distillation. *Int. J. Food Microbiol.* 152 100–106. 10.1016/j.ijfoodmicro.2011.10.006 22056624

[B9] Brigidi P. Swennen E. Vitali B. Rossi M. Matteuzzi D. (2003). PCR detection of bifidobacterium strains and *Streptococcus thermophilus* in feces of human subjects after oral bacteriotherapy and yogurt consumption. *Int. J. Food Microbiol.* 81 203–209. 10.1016/S0168-1605(02)00245-3 12485746

[B10] Charteris W. P. Kelly P. M. Morelli L. Collins J. K. (1998). Antibiotic susceptibility of potentially probiotic *Lactobacillus* species. *J. Food Prot.* 61 1636–1643. 10.4315/0362-028X-61.12.1636 9874341

[B11] Coppola R. Succi M. Tremonte P. Reale A. Salzano G. Sorrentino E. (2005). Antibiotic susceptibility of *Lactobacillus rhamnosus* strains isolated from Parmigiano reggiano cheese. *Lait* 85 193–204. 10.1051/lait:2005007 15727832

[B12] Crittenden R. G. Martinez N. R. Playne M. J. (2003). Synthesis and utilisation of folate by yoghurt starter cultures and probiotic bacteria. *Int. J. Food Microbiol.* 80 217–222. 10.1016/S0168-1605(02)00170-8 12423923

[B13] Curragh H. J. Collins M. A. (1992). High levels of spontaneous drug resistance in *Lactobacillus*. *J. Appl. Microbiol.* 73 31–36.

[B14] De Paula A. T. Jeronymo-Ceneviva A. B. Silva L. F. Todorov S. D. Franco B. D. Penna A. L. (2015). *Leuconostoc mesenteroides* SJRP55: a potential probiotic strain isolated from Brazilian water buffalo mozzarella cheese. *Ann. Microbiol.* 65 899–910. 10.1007/s12602-014-9163-5 24907159

[B15] De Vrese M. Schrezenmeir J. (2008). “Probiotics, prebiotics, and synbiotics,” in *Food Biotechnology, Advances in Biochemical Engineering/Biotechnology* Vol. 111 eds Stahl U. Donalies U. E. B. Nevoigt E. (Berlin: Springer), 1–66. 10.1007/10_2008_097 18461293

[B16] del Carmen S. Miyoshi A. Azevedo V. de LeBlanc A. de M. LeBlanc J. G. (2015). Evaluation of a *Streptococcus thermophilus* strain with innate anti-inflammatory properties as a vehicle for IL-10 cDNA delivery in an acute colitis model. *Cytokine* 73 177–183. 10.1016/j.cyto.2015.02.020 25777482

[B17] EFSA Panel on Additives and Products or Substances used in Animal Feed (2008). Technical guidance for assessing the safety of feed additives for the environment. *EFSA J.* 6:842.

[B18] Ewaschuk J. B. Walker J. W. Diaz H. Madsen K. L. (2006). Bioproduction of conjugated linoleic acid by probiotic bacteria occurs in vitro and in vivo in mice. *J. Nutr.* 136 1483–1487. 10.1093/jn/136.6.1483 16702308

[B19] Favarin L. Laureano-Melo R. Luchese R. H. (2015). Survival of free and microencapsulated bifidobacterium: effect of honey addition. *J. Microencapsul.* 32 329–335. 10.3109/02652048.2015.1017620 25775038

[B20] Gezginc Y. Akyol I. Kuley E. Özogul F. (2013). Biogenic amines formation in *Streptococcus thermophilus* isolated from home-made natural yogurt. *Food Chem.* 138 655–662. 10.1016/J.FOODCHEM.2012.10.138 23265537

[B21] Goldin B. R. (1990). Intestinal microflora: metabolism of drugs and carcinogens. *Ann. Med.* 22 43–48. 10.3109/07853899009147240 1970483

[B22] Halász A. Baráth Á. Simon-Sarkadi L. Holzapfel W. (1994). Biogenic amines and their production by microorganisms in food. *Trends Food Sci. Technol.* 5 42–49. 10.1016/0924-2244(94)90070-90071

[B23] Hirayama K. Rafter J. (2000). The role of probiotic bacteria in cancer prevention. *Microbes Infect.* 2 681–686. 10.1016/S1286-4579(00)00357-9 10884619

[B24] Horne D. W. Patterson D. (1988). *Lactobacillus casei* microbiological assay of folic acid derivatives in 96-well microtiter plates. *Clin. Chem.* 34 2357–2359. 3141087

[B25] Iyer R. Tomar S. K. Uma Maheswari T. Singh R. (2010). *Streptococcus thermophilus* strains: multifunctional lactic acid bacteria. *Int. Dairy J.* 20 133–141. 10.1016/j.idairyj.2009.10.005

[B26] Jacobsen C. N. Nielsen V. R. Hayford A. E. Møller P. L. Michaelsen K. F. Paerregaard A. (1999). Screening of probiotic activities of forty-seven strains of *Lactobacillus* spp. by in vitro techniques and evaluation of the colonization ability of five selected strains in humans. *Appl. Environ. Microbiol.* 65 4949–4956. 10543808 10.1128/aem.65.11.4949-4956.1999 PMC91666

[B27] Jeronymo-Ceneviva A. B. de Paula A. T. Silva L. F. Todorov S. D. Franco B. D. G. Penna A. L. (2014). Probiotic properties of lactic acid bacteria isolated from water-buffalo mozzarella cheese. *Probiotics Antimicrob. Proteins* 6 141–156. 10.1007/s12602-014-9166-2 25117002

[B28] Jiang W. G. Sanders A. J. Katoh M. Ungefroren H. Gieseler F. Prince M. (2015). Tissue invasion and metastasis: molecular, biological and clinical perspectives. *Semin. Cancer Biol.* 35 S244–S275. 10.1016/J.SEMCANCER.2015.03.008 25865774

[B29] FAO (2002). WHO Working Group Report on Drafting Guidelines for the Evaluation of Probiotics in Food. London: WHO, 30.

[B30] Khali R. K. (2009). Evidence for probiotic potential of a capsular-producing *Streptococcus thermophilus* CHCC 3534 strain. *Afr. J. Microbiol. Res.* 3 27–34. 19469286

[B31] Konings E. J. M. Roomans H. H. S. Dorant E. Goldbohm R. A. Saris W. H. M. van den Brandt P. A. (2001). Folate intake of the dutch population according to newly established liquid chromatography data for foods–. *Am. J. Clin. Nutr.* 73 765–776. 10.1093/ajcn/73.4.765 11273852

[B32] Kumar M. Verma V. Nagpal R. Kumar A. Behare P. V. Singh B. (2012). Anticarcinogenic effect of probiotic fermented milk and chlorophyllin on aflatoxin-B 1-induced liver carcinogenesis in rats. *Br. J. Nutr.* 107 1006–1016. 10.1017/S0007114511003953 21816119

[B33] Ladero V. Martín M. C. Redruello B. Mayo B. Flórez A. B. Fernández M. (2015). Genetic and functional analysis of biogenic amine production capacity among starter and non-starter lactic acid bacteria isolated from artisanal cheeses. *Eur. Food Res. Technol.* 241 377–383. 10.1007/s00217-015-2469-z

[B34] Lick S. Drescher K. Heller K. J. (2001). Survival of *Lactobacillus delbrueckii* subsp. bulgaricus and *Streptococcus thermophilusin* the terminal ileum of fistulated göttingen minipigs. *Appl. Environ. Microbiol.* 67 4137–4143. 10.1128/AEM.67.9.4137-4143.2001 11526016 PMC93140

[B35] Mamianetti A. Garrido D. Carducci C. N. Cristina Vescina M. (1999). Fecal bile acid excretion profile in gallstone patients. *Medicina* 59 269–273. 10451567

[B36] Maragkoudakis P. A. Nardi T. Bovo B. D’Andrea M. Howell K. S. Giacomini A. (2013). Biodiversity, dynamics and ecology of bacterial community during grape marc storage for the production of grappa. *Int. J. Food Microbiol.* 162 143–151. 10.1016/j.ijfoodmicro.2013.01.005 23416549

[B37] Mater D. D. G. Bretigny L. Firmesse O. Flores M. J. Mogenet A. Bresson J. L. (2005). *Streptococcus thermophilus* and *Lactobacillus delbrueckii* subsp. bulgaricus survive gastrointestinal transit of healthy volunteers consuming yogurt. *FEMS Microbiol. Lett.* 250 185–187. 10.1016/j.femsle.2005.07.006 16099606

[B38] Mete A. Coşansu S. Demirkol O. Ayhan K. (2017). Amino acid decarboxylase activities and biogenic amine formation abilities of lactic acid bacteria isolated from shalgam. *Int. J. Food Prop.* 20 171–178. 10.1080/10942912.2016.1152479

[B39] Morgulis A. Coulouris G. Raytselis Y. Madden T. L. Agarwala R. Schäffer A. A. (2008). Database indexing for production MegaBLAST searches. *Bioinformatics* 24 1757–1764. 10.1093/bioinformatics/btn322 18567917 PMC2696921

[B40] Mosmann T. (1983). Rapid colorimetric assay for cellular growth and survival: application to proliferation and cytotoxicity assays. *J. Immunol. Methods* 65 55–63. 10.1016/0022-1759(83)90303-4 6606682

[B41] Motevaseli E. Dianatpour A. Ghafouri-Fard S. (2017). The role of probiotics in cancer treatment: emphasis on their in vivo and in vitro anti-metastatic effects. *Int. J. Mol. Cell. Med.* 6 66–76. 10.22088/acadpub.BUMS.6.2.1 28890883 PMC5581548

[B42] Oh Y. J. Jung D. S. (2015). Evaluation of probiotic properties of *Lactobacillus* and pediococcus strains isolated from Omegisool, a traditionally fermented millet alcoholic beverage in Korea. *LWT Food Sci. Technol.* 63 437–444. 10.1016/j.lwt.2015.03.005

[B43] Otto R. Brink B. Veldkamp H. Konings W. N. (1983). The relation between growth rate and electrochemical proton gradient of Streptococcus cremoris. *FEMS Microbiol. Lett.* 16 69–74. 10.1111/j.1574-6968.1983.tb00261.x

[B44] Pedrosa M. C. Golner B. B. Goldin B. R. Barakat S. Dallal G. E. Russell R. M. (1995). Survival of yogurt-containing organisms and *Lactobacillus gasseri* (ADH) and their effect on bacterial enzyme activity in the gastrointestinal tract of healthy and hypochlorhydric elderly subjects. *Am. J. Clin. Nutr.* 61 353–359. 10.1093/ajcn/61.2.353 7840074

[B45] Peres C. M. Alves M. Hernandez-Mendoza A. Moreira L. Silva S. Bronze M. R. (2014). Novel isolates of lactobacilli from fermented portuguese olive as potential probiotics. *LWT Food Sci. Technol.* 59 234–246. 10.1016/j.lwt.2014.03.003

[B46] Piatek J. Gibas-Dorna M. Olejnik A. Krauss H. Wierzbicki K. Zukiewicz-Sobczak W. (2012). The viability and intestinal epithelial cell adhesion of probiotic strain combination-in vitro study. *Ann. Agric. Environ. Med.* 19 99–102. 22462453

[B47] Pieniz S. Andreazza R. Anghinoni T. Camargo F. Brandelli A. (2014). Probiotic potential, antimicrobial and antioxidant activities of *Enterococcus durans* strain LAB18s. *Food Control* 37 251–256. 10.1016/j.foodcont.2013.09.055

[B48] Rao D. R. Reddy A. V. Pulusani S. R. Cornwell P. E. (1984). Biosynthesis and utilization of folic acid and vitamin B12 by lactic cultures in skim milk. *J. Dairy Sci.* 67 1169–1174. 10.3168/jds.S0022-0302(84)81419-8

[B49] Rossi F. Gardini F. Rizzotti L. La Gioia F. Tabanelli G. Torriani S. (2011). Quantitative analysis of histidine decarboxylase gene (hdcA) transcription and histamine production by *Streptococcus thermophilus* PRI60 under conditions relevant to cheese making. *Appl. Environ. Microbiol.* 77 2817–2822. 10.1128/AEM.02531-10 21378060 PMC3126354

[B50] Ruas-Madiedo P. Gueimonde M. De Los Reyes-Gavilan C. G. Salminen S. (2006). Effect of exopolysaccharide isolated from “viili” on the adhesion of probiotics and pathogens to intestinal mucus. *J. Dairy Sci.* 89 2355–2358. 10.3168/jds.S0022-0302(06)72307-4 16772550

[B51] Salminen S. Isolauri E. (2006). Intestinal colonization, microbiota, and probiotics. *J. Pediatr.* 149 S115–S120. 10.1016/j.jpeds.2006.06.062

[B52] Santos M. H. S. (1996). Biogenic amines: their importance in foods. *Int. J. Food Microbiol.* 29 213–231. 10.1016/0168-1605(95)00032-1 8796424

[B53] Senok A. C. Ismaeel A. Y. Botta G. A. (2005). Probiotics: facts and myths. *Clin. Microbiol. Infect.* 11 958–966. 10.1111/j.1469-0691.2005.01228.x 16307549

[B54] Sybesma W. Starrenburg M. Tijsseling L. Hoefnagel M. H. N. Hugenholtz J. (2003). Effects of cultivation conditions on folate production by lactic acid bacteria. *Appl. Environ. Microbiol.* 69 4542–4548. 10.1128/AEM.69.8.4542-4548.2003 12902240 PMC169137

[B55] Tallon R. Arias S. Bressollier P. Urdaci M. C. (2007). Strain-and matrix-dependent adhesion of *Lactobacillus plantarum* is mediated by proteinaceous bacterial compounds. *J. Appl. Microbiol.* 102 442–451. 10.1111/j.1365-2672.2006.03086.x 17241350

[B56] Tarrah A. Noal V. Giaretta S. Treu L. da Silva Duarte V. Corich V. (2018a). Effect of different initial pH on the growth of Streptococcus macedonicus and *Streptococcus thermophilus* strains. *Int. Dairy J.* 86 65–68. 10.1016/J.IDAIRYJ.2018.07.003 29960771

[B57] Tarrah A. Noal V. Treu L. Giaretta S. da Silva Duarte V. Corich V. (2018b). Short communication: Comparison of growth kinetics at different temperatures of Streptococcus macedonicus and *Streptococcus thermophilus* strains of dairy origin. *J. Dairy Sci.* 101 7812–7816. 10.3168/JDS.2018-14731 29960771

[B58] Tarrah A. Treu L. Giaretta S. Duarte V. Corich V. Giacomini A. (2018c). Differences in carbohydrates utilization and antibiotic resistance between Streptococcus macedonicus and *Streptococcus thermophilus* strains isolated from dairy products in Italy. *Curr. Microbiol.* 10.1007/s00284-018-1528-1527 [Epub ahead of print], 29916034

[B59] Thomas M. Wrzosek L. Ben-Yahia L. Noordine M.-L. Gitton C. Chevret D. (2011). Carbohydrate metabolism is essential for the colonization of *Streptococcus thermophilus* in the digestive tract of gnotobiotic rats. *PLoS One* 6:e28789. 10.1371/journal.pone.0028789 22216112 PMC3245227

[B60] Treu L. Vendramin V. Bovo B. Campanaro S. Corich V. (2014a). Genome sequences of four Italian *Streptococcus thermophilus* strains. *Genome Announc.* 2 2008–2009. 10.1128/genomeA.00126-14.Copyright 24625867 PMC3953188

[B61] Treu L. Vendramin V. Bovo B. Campanaro S. Corich V. Giacomini A. (2014b). Genome sequences of four Italian *Streptococcus thermophilus* strains of dairy origin. *Genome Announc* 2:e00126-14. 10.1128/genomeA.00126-114 24625867 PMC3953188

[B62] Treu L. Vendramin V. Bovo B. Campanaro S. Corich V. Giacomini A. (2014c). Genome sequences of *Streptococcus thermophilus* strains MTH17CL396 and M17PTZA496 from fontina, an Italian PDO cheese. *Genome Announc* 2:e00067-14. 10.1128/genomeA.e00067-14 24526643 PMC3924375

[B63] Treu L. Vendramin V. Bovo B. Campanaro S. Corich V. Giacomini A. (2014d). Whole-genome sequences of *Streptococcus thermophilus* strains TH1435 and TH1436, isolated from raw goat milk. *Genome Announc.* 2:e01129-13. 10.1128/genomeA.01129-1113 24435859 PMC3894273

[B64] Valentini L. Pinto A. Bourdel-Marchasson I. Ostan R. Brigidi P. Turroni S. (2015). Impact of personalized diet and probiotic supplementation on inflammation, nutritional parameters and intestinal microbiota – The “RISTOMED project”: Randomized controlled trial in healthy older people. *Clin. Nutr.* 34 593–602. 10.1016/j.clnu.2014.09.023 25453395

[B65] Vendramin V. Treu L. Campanaro S. Lombardi A. Corich V. Giacomini A. (2017). Genome comparison and physiological characterization of eight *Streptococcus thermophilus* strains isolated from Italian dairy products. *Food Microbiol.* 63 47–57. 10.1016/j.fm.2016.11.002 28040181

[B66] Wayne P. A. (2002). National committee for clinical laboratory standards. *Perform. Stand. Antimicrob. Disc Susceptibility Test.* 12 1–53.

[B67] Wu Q. Cheung C. K. W. Shah N. P. (2015). Towards galactose accumulation in dairy foods fermented by conventional starter cultures: challenges and strategies. *Trends Food Sci. Technol.* 41 24–36. 10.1016/j.tifs.2014.08.010

