# Supplementary material for: In vitro Probiotic Potential and Anti-cancer Activity of Newly Isolated Folate-Producing Streptococcus thermophilus Strains
Source: Front Microbiol. 2018 Sep 19;9:2214. doi: 10.3389/fmicb.2018.02214 (PMC6156529; doi:10.3389/fmicb.2018.02214)
Supplement: Supplementary file 1 [file Data_Sheet_1.docx]

**Fig S1**. Results of the test for production of histamine and tyramine A) *S. thermophilus* TH1436; B) *thermophilus* MTH17CL396; C) *thermophilus* M17PTZA496; D) *thermophilus* TH982. Purple colored tubes are positive. Control tubes do not contain the substrate for the production of the amines. The strains not displayed behaved as C and D (no production).


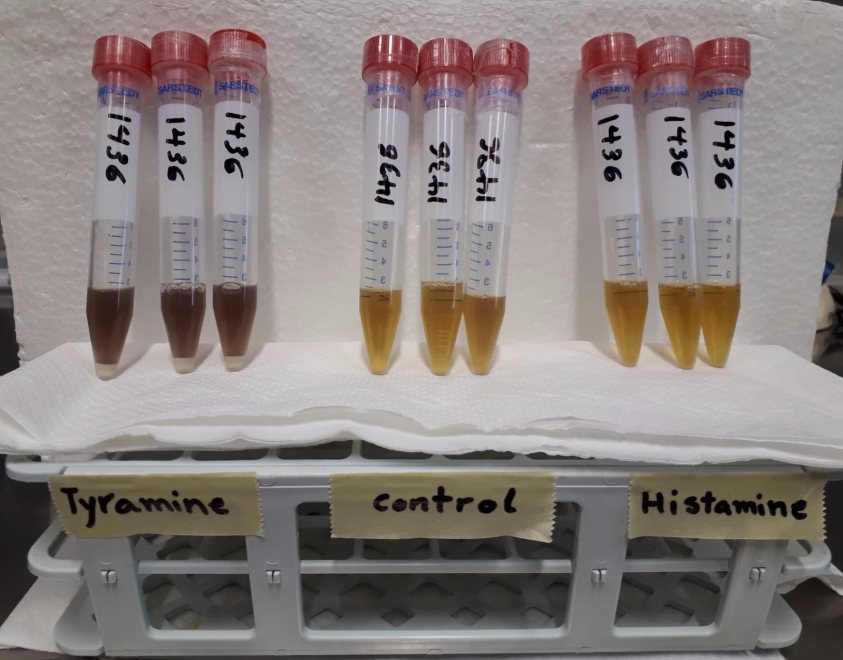

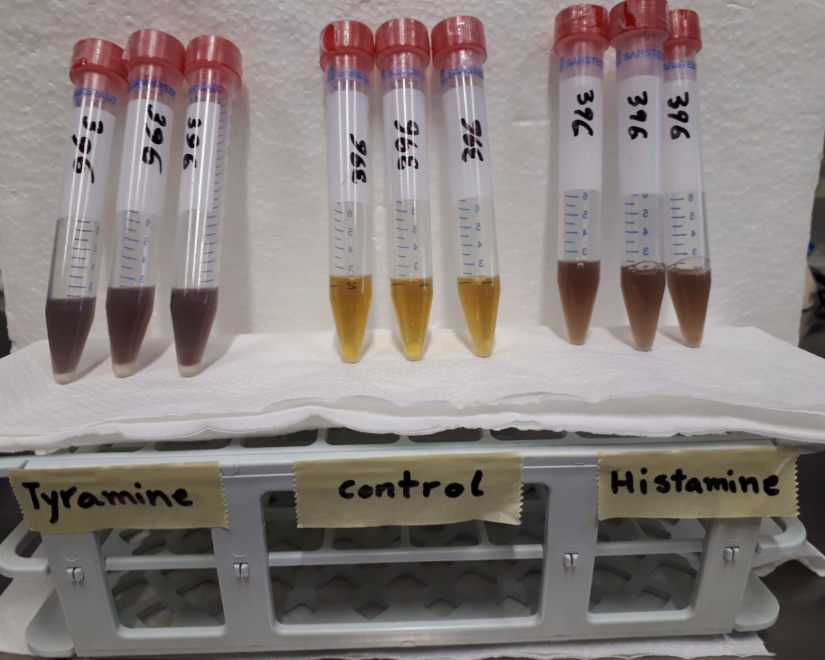

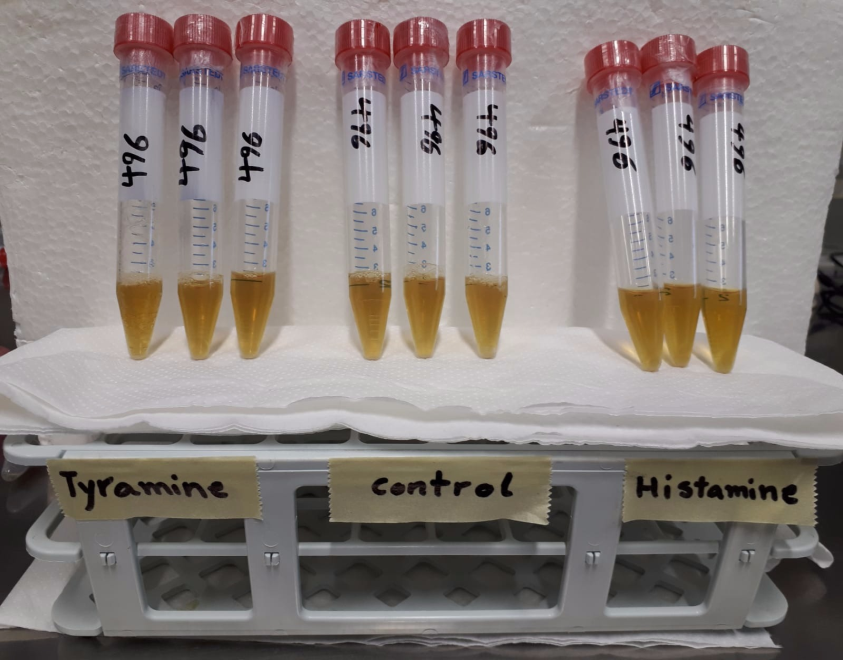

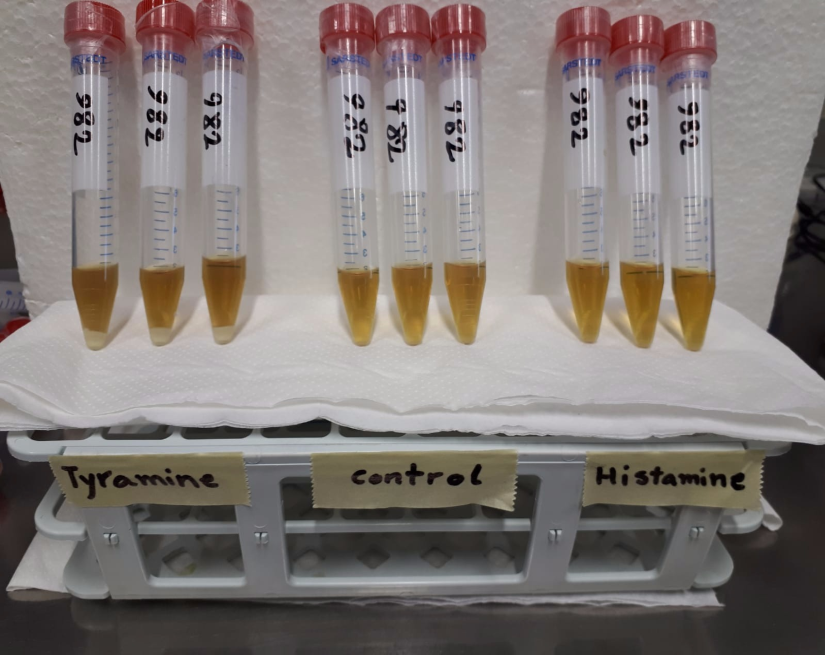


**A**

**B**

**C**

**D**


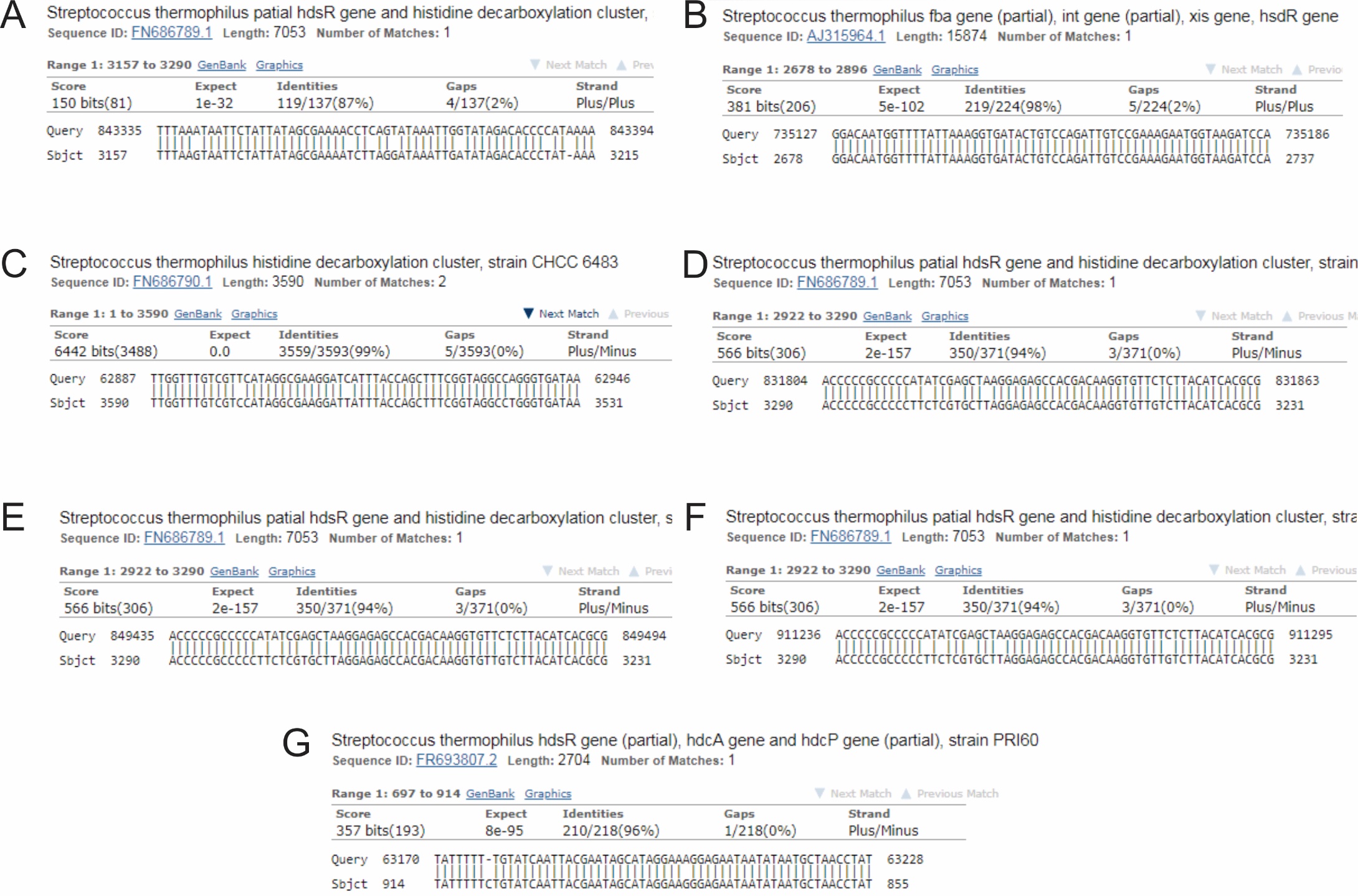


**Fig. S2**. BLASTn analysis of genes involved in histamine production in *S. thermophilus* strains, using the histidine decarboxylation cluster *(hdc*) of *S. thermophilus* CHCC6483 (Accession number FN686790.1) as query sequence. A) *S. thermophilus* M17PTZA496; B) *S. thermophilus* MTH17CL396; C) *S. thermophilus* TH1435; D) *S. thermophilus* TH1436; E) *S. thermophilus* TH982; F) *S. thermophilus* 1F8CT. *S. thermophilus* TH985 did not show any sequence similarity to the *hdc* cluster. The sequences of the *S. thermophilus* strains tested are indicated as “Query”.
